# DNA damage response and repair data with pharmacological modulators of Tousled

**DOI:** 10.1016/j.dib.2016.03.075

**Published:** 2016-03-30

**Authors:** Prakash Srinivasan Timiri Shanmugam, Renjith P. Nair, Arrigo DeBenedetti, Gloria Caldito, Fleurette Abreo, Gulshan Sunavala-Dossabhoy

**Affiliations:** a Department of Biochemistry and Molecular Biology, USA; b Department of Computational Biology and Bioinformatics, USA; c Department of Pathology, LSU Health Sciences Center, Shreveport, LA, USA

**Keywords:** Tousled, Ionizing radiation, DNA repair, Gallic acid, Thioridazine

## Abstract

Human Tousled kinase 1 (TLK1) plays an important role in chromatin remodeling, replication, and DNA damage response and repair. TLK1 activity is immediately, but transiently, downregulated after genotoxic insult, and its recovery is important for exit from checkpoint arrest and cell survival after radiation. The data in this article compliments research presented in the paper titled, “Tousled kinase activator, gallic acid, promotes DNA repair and suppresses radiation cytotoxicity in salivary gland cells” [1]. The identification of small molecule activators and inhibitors of TLK1 provided an opportunity to pharmacologically alter the protein׳s activity to elucidate its role in DNA damage response pathways. TLK1 effectors, gallic acid (GA) and thioridazine (THD) activate and inhibit the kinase, respectively, and the data report on the impact of these compounds and the significance of TLK1 to DNA break repair and the survival of human salivary acinar cells.

## **Specifications Table**

**Table:** 

Subject area	*Molecular biology*
More specific subject area	*DNA break repair*
Type of data	*Image, graph, figure*
How data was acquired	*Olympus Provis AX70 microscope, BD LSR II flow cytometer, BioTek Synergy 4 hybrid microplate reader.*
Data format	*Raw*
Experimental factors	*Cells were treated with 10* *µM THD 1* *h before drug washout and irradiation, whereas cell treatment with 50* *µM GA followed 30 minutes after radiation and extended up to indicated experimental time points or 16* *h before placement in drug-free medium.*
Experimental features	*NS-SV-AC and stably transfected shRNA TLK1 cells were grown in complete KGM-2 medium. Cells were treated with drug before or after radiation, and cells were analyzed by colony formation assays, single cell alkaline gel electrophoreses, or Annexin V staining at indicated times. Radiation-generated reactive oxygen species was analyzed using CM-H2DCFDA. Repair of restriction enzyme (I SceI)- induced double strand break was studied in HEK293-PC222 cells. Cell population that became RFP positive was quantified by FACS analyses.*
Data source location	*Shreveport, LA*
Data accessibility	*Data accessible in the article*

## **Value of the data**

• Influence of small molecule TLK1 effectors on cell survival highlight their clinical utility in altering tissue response to radiation.
• Studies that assess synthetic lethality of TLK1 inhibitors and genotoxic therapeutics can initiate the development of more effective cancer treatment regimens.
• Enhanced TLK1 activity leads to improved DNA break repair kinetics, and an examination of TLK1-dependent dynamics at DNA breaks may lead to the identification of its yet unknown protein targets.

## 1 Data

The data in the article demonstrate the effects of small molecule modulators of TLK1 activity in DNA damage response to and repair of radiation-induced DNA breaks. Unrepaired DNA breaks activate apoptosis, and the consequential effect of TLK1 function on cell survival and programmed cell death was demonstrated. The repair of DNA double-strand breaks predominantly occurs by non-homologous end joining (NHEJ), and using a cell-based reporter assay we reported the effect of TLK1 function on NHEJ repair of I-SceI-induced chromosomal breaks.

## 2 Experimental design, materials and methods

### 2.1 Cell culture

The acinar cells of the salivary glands are fluid-producing cells that are highly sensitive to radiation. Their inadvertent damage and resultant death during head and neck radiotherapy results in an irreversible decline in salivary gland function [2]. Preservation of these cells is critical to suppressing salivary hypofunction. Human submandibular acinar cell line, NS-SV-AC, (a kind gift from Dr. Masayuki Azuma, University of Tokushima School of Dentistry, Tokushima, Japan) was, therefore, used in the study. Cells were grown at 37 °C in complete keratinocyte growth medium 2 (KGM-2; Lonza) supplemented with antibiotic/antimycotic (Invitrogen). Knockdown of TLK1 was achieved by transfecting cells with human 29-mer TLK1 shRNA (ATTACTTCATCTGCTTGGTAGAGGTGGCT; Origene), and selecting a multiclonal cell population under puromycin (1 µg/ml) challenge. Stock solutions (10 mM) of GA and THD (Sigma) were made in water before use.

### 2.2 Colony survival assay

Previous studies demonstrated a role of TLK1 in salivary gland survival against radiation [[3], [4], [5]]. To explore the relationship between protein activity and cell survival, small molecules that modulate TLK1 were examined. The experimental layout to study the effect of TLK1 inhibition by THD is shown in Fig. S1A. NS-SV-SC cells were treated, or not, with 10 µM THD 1 h before trypsinizing and resuspending cells at 1×10^5^ cells/ml. They were irradiated at 0, 2, 4, 8, or 12 Gy, and seeded in triplicates at different densities in 12-well plates. On day 10 post-radiation, cell colonies were stained with crystal violet and counted. The effect of short-term TLK1 inhibition on cell viability after radiation is demonstrated in the colony formation assay graph (Fig. S1B).

### 2.3 Comet assay

A role of TLK1 in DNA double-strand break repair was proposed before [6], [7]. To study the influence of activators and inhibitors of TLK1 on DNA repair, Trevigen Comet Assay kit (Trevigen) was used to analyze radiation-induced single and double-stranded DNA breaks. Single-cell electrophoresis in alkaline conditions was conducted as per the manufacturer׳s instructions. Sub-confluent cell cultures in 35 mm plates were exposed to 8 Gy on ice. Cells were treated with THD (10 µM), 1 h, prior to radiation, or exposed to GA (50 µM) at 0.5 h after it. Cells were collected immediately after radiation or allowed to recover for indicated times at 37 °C. Cells were thereafter trypsinized, washed and resuspended in Ca^+2^ and Mg^+2^-free ice cold PBS, pH 7.4. Cell aliquots were mixed with low melting agarose at 37 °C, and 50 µl of mix was evenly spread in each well of the comet slide. The agarose was allowed to set before placing the slides in prechilled lysis solution (2.5 M NaCl, 100 mM EDTA, 10 mM Tris-base, 1% sodium lauryl sarcosinate, 1% Triton X-100, pH 10) overnight at 4 °C. DNA was denatured in alkaline solution (300 mM NaOH, 1 mM EDTA, pH>13) for 20 min in the dark at room temperature before electrophoresis (1 V/cm, 30 mA) at 4 °C for 30 min in prechilled alkaline solution. Slides were rinsed in distilled water twice followed by 70% ethanol before drying and staining them with 100 µl propidium iodide (Sigma; 10 µg/ml in PBS, pH 7.4). Images were captured using a TRITC filter mounted on an Olympus epifluorescence microscope, and the DNA tail length and tail moment were measured using CASP software (http://www.casp.of.pl). The effect of shRNA–TLK1 knockdown on DNA break repair is presented (Fig. S1C), and the results compared to parental NS-SV-AC and derived shRNA-TLK1 knockdown cells exposed to GA (Fig. S1D).

### 2.4 Immunoblotting

The involvement of TLK1 in genotoxic stress-induced checkpoint arrest was shown previously [8], [9]. TLK1 is rapidly, but transiently, suppressed by Chk1 immediately after DNA damage. To evaluate the effects of pharmacological agents in cellular response to radiation, NS-SV-AC or shRNA–TLK1 cells were exposed to 8 Gy and treated thereafter with GA (50 µM) for up to 16 h. Cells were collected at indicated times, and total sample protein (30 μg) was electrophoresed on 10% SDS-polyacrylamide gel. Proteins were blotted on PVDF membrane (Bio-Rad), and the membrane was immersed in 5% nonfat dry milk for 1 h. The blot was reacted overnight at 4 °C with primary antibody to phospho-T68 Chk2 (Cell Signaling), phospho-S216 Cdc25C (Abcam), phospho-Y15 Cdk1 (Abcam), or β-actin (Sigma) at 1:1000 dilution in TBS / 0.2% Tween 20. The blot was washed thrice in TBS/ 0.2% Tween 20 before reacting it with corresponding HRP-conjugated secondary antibody. DNA damage response proteins involved in radiation-induced G2 checkpoint control were visualized after reaction with a chemiluminescent HRP substrate (Thermo Scientific). The phosphorylation status of checkpoint kinase, Chk2 (T68) and G2 checkpoint proteins, Cdc25C (S216) and Cdk1 (Y15) in untreated and GA-treated cells is shown (Fig. S2A). For comparison, immunoblots of shRNA–TLK1 knockdown cell lysates are included (Fig. S2B).

### 2.5 Annexin V/Propidium iodide staining

DNA repair is fundamental to survival, and the effect of TLK1 compounds on radiation-induced cell death was examined. NS-SV-AC were labeled using FITC Annexin V Apoptosis Detection Kit (BD Pharmingen) as per the manufacturer׳s protocol. Cells were treated with THD prior or with GA after exposure to 8 Gy. GA was washed out after 16 h, and cells were placed in fresh medium before collecting them at 48 h. Cells were washed with cold PBS and resuspended at 1×10^6^ cells/ml in binding buffer (0.01 M HEPES, pH 7.4, 140 mM NaCl, 2.5 mM CaCl_2_). Cells were incubated in the dark for 15 min after the addition of Annexin V-FITC and propidium iodide (5 µl each) to 100 µl of cell suspension. The cell mix was diluted with 400 µl binding buffer, and the samples were analyzed using a BD LSR II flow cytometer. Cells that were unstained, treated with Annexin V-FITC alone, or treated with PI alone were used as controls for quadrant set-up. The distribution of cells by biparametric annexin-V and PI staining is presented (Fig. S3).

### 2.6 Measurement of radiation-generated intracellular ROS

GA is a known anti-oxidant and can influence the results of DNA repair and cell survival assays. Therefore, a fluorescent probe, CM-H2DCFDA (Invitrogen), was used for measurement of radiation-generated intracellular reactive-oxygen species (ROS). NS-SV-AC cells were detached and resuspended in medium at 2×10^5^ cells/ml. 5 ml of cell suspension was incubated with 5 µM CM-H2DCFDA for 30 min at 37 °C in a humidified CO_2_ incubator. Cells were centrifuged and unincorporated probe was washed out. The cell suspension was then irradiated (8 Gy) before plating 2×10^4^ cells/well in 96 well plates. GA was added 30 min later, and fluorescence was analyzed at indicated times. The effect of GA on levels of intracellular ROS was thereafter plotted (Fig. S4).

### 2.7 Non-homologous DNA repair assay

To conclusively demonstrate that the ROS scavenging property of GA was not a predominant player in DNA repair outcome, repair of endonuclease-cleaved DNA was studied in HEK293T PC222 cells (a kind gift from Dr. Kum Kum Khanna, Queensland Institute of Medical Research, Australia). HEK293T PC222 cells harbor a chromosomally integrated GFP-iRFP cassette for the examination of non-homologous end joining (NHEJ, Fig. S5A). To conduct the assay, cells were seeded in 60 mm plates and allowed to adhere overnight. Before transfection, cells were either pretreated, or not, with THD (10 µM, 1 h). The drug was washed out, and a transfection mix with OPTI-MEM diluted I-SceI plasmid (10 µg) and Lipofectamine 2000 was layered on cells. Six hours after transfection, the cells were detached and seeded at equal density in three 60 mm plates to which vehicle or GA (50 µM) was added. GA was washed out after 16 h, and cells cultured in drug-free medium up to 72 h. Cells were lifted-off plates, and single cell suspension was run through a flow cytometer. Data of 50,000 viable single cells was acquired using appropriate lasers. The population of fluorescing RFP cells was reported as percent NHEJ, which reflected on TLK1׳s contribution to the process (Fig. S5B).

## Footnotes

Appendix A Supplementary data associated with this article can be found in the online version at 10.1016/j.dib.2016.03.075.
